# MSCs’ conditioned media cytokine and growth factor profiles and their impact on macrophage polarization

**DOI:** 10.1186/s13287-023-03381-w

**Published:** 2023-05-25

**Authors:** Maria Peshkova, Alexander Korneev, Shakir Suleimanov, Irina I. Vlasova, Andrey Svistunov, Nastasia Kosheleva, Peter Timashev

**Affiliations:** 1 World-Class Research Center “Digital Biodesign and Personalized Healthcare”, Sechenov University, Moscow, Russia 119991; 2 Institute for Regenerative Medicine, Sechenov University, Moscow, Russia 119991; 3 Laboratory of Clinical Smart Nanotechnologies, Sechenov University, Moscow, Russia 119991; 4 Laboratory of the Polymers Synthesis for Medical Applications, Sechenov University, Moscow, Russia 119991; 5 Sechenov First Moscow State Medical University, Moscow, Russia 119991; 6 FSBSI Institute of General Pathology and Pathophysiology, Moscow, Russia 125315

**Keywords:** Mesenchymal stromal cells, Conditioned media, Secretome, Cytokines, Immunomodulation, Macrophages

## Abstract

**Background:**

There is a growing body of evidence that multipotent mesenchymal stromal cells’ (MSCs’) remarkable therapeutic potential is attributed not only to their differentiation and regenerative capacity, but also to the paracrine effect, underlying their immunomodulatory properties. MSCs’ secretome (i.e., cytokines, growth factors, and extracellular vesicles) is therefore increasingly discussed in the context of their ability to modulate inflammatory response and promote regeneration. There is evidence that 2D or 3D culturing conditions have an impact on the cells’ secretome, and here we aimed to compare the secretion of cytokines and growth factors in human MSCs from different sources cultured in 2D and 3D conditions and assess their effect on human macrophages polarization in vitro.

**Methods:**

MSCs were derived from human adipose tissue, bone marrow, gingiva, placenta, and umbilical cord, cultured as monolayers or as cell spheroids. Their cytokine profiles were analyzed, and data standardization was carried out using a *z*-score. Human peripheral blood mononuclear cells-derived macrophages were then treated with umbilical cord-derived MSCs’ conditioned media and their effect on macrophages polarization was assessed.

**Results:**

Our findings suggest that umbilical cord-derived MSCs’ conditioned media demonstrated the highest cytokine and growth factor levels and despite mostly pro-inflammatory cytokine profile were able to promote anti-inflammatory macrophage polarization.

**Conclusions:**

Umbilical cord-derived MSCs’ conditioned media hold great potential for therapeutic use, demonstrating significant anti-inflammatory effect on human macrophages.

**Supplementary Information:**

The online version contains supplementary material available at 10.1186/s13287-023-03381-w.

## Background

Multipotent mesenchymal stromal cells (MSCs) are an extensively researched yet still understudied tool for treating a wide range of pathological conditions, one particular area of research interest being their immunomodulatory properties.

MSCs are reported to contribute both to the inflammation initiation and to its resolution via complex cross talk with immune cells, most importantly macrophages [1]. Non-activated M0 macrophages can be activated toward pro-inflammatory (M1) and anti-inflammatory (M2) phenotypes, thus playing important and diverse roles at all stages of inflammatory response. Although this binary classification does not take into account the variety of subcategories of macrophages phenotypes [2], it can be useful for studying the mechanisms of macrophage viability and activity regulation [3].

Activation of macrophages into different states is a finely tuned process essential for proper resolution of inflammation and tissue repair. MSCs have been shown to suppress both the transition of human macrophages from the M0 to M1 state and the pro-inflammatory activity of macrophages already polarized into the M1 state [4]. MSCs were also reported to support monocyte survival skewing their polarization toward a M2-like phenotype through a prostaglandin E2-dependent mechanism [5].

Given the minimal criteria for defining MSCs [6], it may seem that MSCs derived from different sources should exhibit equivalent properties. However, there is evidence that MSCs from each niche are characterized by some unique features, suggesting that the choice of the MSCs source in every particular case should be given due consideration.

Thus, bone marrow is the first established and still one of the most commonly used sources of MSCs [7]. Bone marrow-derived MSCs (BM-MSCs) are characterized by increased osteogenic and chondrogenic differentiation potential [8]; however, relatively low cell yield and high invasiveness of the harvesting procedure [9] have urged the quest for alternative MSCs sources. Given MSCs differentiation directions, adipose tissue was considered another valid source of MSCs (AT-MSCs). Being harvested in a less invasive manner than bone marrow [10], it was also reported to give a higher MSCs yield [11]. As MSCs research expanded, more sources for their harvesting were proposed. For example, dental pulp and gingiva were recognized as sources of MSCs with unique neuroectodermal features due to their neural crest origin [12, 13]. Neonatal tissues, such as umbilical cord and placenta, have gained particular interest, giving a chance to harvest MSCs in a noninvasive manner. Furthermore, MSCs derived from neonatal tissues were reported to have improved proliferative and engraftment capacities compared to MSCs from adult tissues [14].

However, certain concerns regarding MSCs clinical application have arisen in the past years. Leaving aside tumorigenicity risks, MSCs demonstrate relatively low survival rates post administration; moreover, their behavior in vivo, including surface molecule expression [15] and paracrine activity [16], is highly unpredictable [17, 18], being strongly influenced by the microenvironment [16]. In this light, the use of MSCs’ secretome rather than MSCs themselves seems an attractive option, since it has long been argued that MSCs paracrine activity underlying their immunomodulatory properties makes an even greater contribution to their therapeutic potential than their differentiation capacities [19].

MSCs conditioned media (CM) contain their whole secretome including cytokines, growth factors, and extracellular vesicles (EVs), and are reported to show good results in treating various pathological conditions in vivo [20]. Predictably, MSCs niches affect their secretomes and therefore their immunomodulatory effects [21].

Here, we aimed to compare the secretomes of AT-MSCs, BM-MSCs, gingiva-derived MSCs (G-MSCs), placenta-derived MSCs (PL-MSCs), and umbilical cord-derived MSCs (UC-MSCs) cultured as monolayers (2D) and as cell spheroids (3D), since there is evidence that culturing conditions also have an impact on the cells’ secretome [22]. We have analyzed the expression of 41 cytokines and growth factors in the CM samples taken on the 3rd day of culturing and assessed the MSCs-derived CM effect on macrophages polarization in vitro.

## Materials and methods

### MSCs sources

Human BM-MSCs were obtained from the Biobank of the Institute for Regenerative medicine (Sechenov University, Moscow, Russia). Human AT-MSCs, G-MSCs, PL-MSCs, and UC-MSCs were isolated from adipose tissue, gingiva, placenta, and umbilical cord, respectively. In accordance with the Declaration of Helsinki, all the tissue samples were collected after the donor signed an informed consent form approved by the local ethics committee of Sechenov University.

#### AT-MSCs isolation

For AT-MSCs isolation, the previously reported protocols [23, 24] were adjusted as follows: abdominal subcutaneous adipose tissue biopsy samples were thoroughly washed with Hanks’ solution (BioloT, Russia) supplemented with Diflucan (Pfizer, USA), gentamicin (50 μg/ml, PanEco, Russia), and penicillin/streptomycin (100 U/ml/100 µg/ml, PanEco, Russia). Each sample was minced into small (~ 1 mm^3^) pieces with scissors and transferred into conical tubes. A fourfold volume of 0.1% collagenase type I solution (PanEco, Russia) in Dulbecco’s Modified Eagle’s Medium (DMEM)/F12 supplemented with 2 mM l-glutamine (BioloT, Russia) and 50 mg/ml gentamicin was added to each tube, and enzymatic dissociation was performed for 20 min at 37 °C. Enzymatic digestion was blocked by adding threefold volume of DMEM/F12 supplemented with 2 mM l-glutamine (BioloT, Russia), 10% fetal bovine serum (FBS) (Thermo Fisher, USA), and penicillin/streptomycin (100 U/ml/100 µg/ml, PanEco, Russia). The tubes were then centrifuged at 100*g* for 10 min at room temperature and the supernatant was discarded. The cell pellet was resuspended in DMEM/F12 supplemented with 2 mM l-glutamine (BioloT, Russia), 10% FBS (Thermo Fisher, USA), and penicillin/streptomycin (100 U/ml/100 µg/ml, PanEco, Russia) and seeded on Petri dishes.

#### PL-MSCs isolation

PL-MSCs isolation was performed as follows: placenta tissue samples were washed with Hanks’ solution (BioloT, Russia) supplemented with 50 U/ml amphotericin and 100 U/ml penicillin for 12 h. Each sample was minced into small (~ 3 mm^3^) pieces, and enzymatic dissociation in a 0.15% collagenase I solution (Serva, Germany) was performed at 37 °C for 30 min with constant agitation on Mini Rocker-Shaker (Biosan, Latvia). We then added Hanks’ solution to the suspension, passed it through a 100-μm filter (Becton Dickinson, USA), and centrifuged at 300*g* for 10 min. The cell pellet was resuspended in DMEM/F12 supplemented with 2 mM l-glutamine (BioloT, Russia), 10% FBS (Hyclone, Germany), 5 mM HEPES (Biomedicals, USA), 100 U/ml penicillin, and 50 µg/ml amphotericin and seeded on Petri dishes.

#### G-MSCs isolation

G-MSCs were isolated as follows: gingival tissue samples were washed in α-MEM solution (Sigma, USA) supplemented with gentamicin (50 µg/ml, PanEco, Russia) for 12 h. Each sample was minced into ~ 1 mm^3^ fragments with sterile eye scissors, and enzymatic dissociation in 0.05% collagenase II solution (Sigma, USA) in α-MEM supplemented with 10% FBS (Thermo Fisher, USA) and gentamicin (50 µg/ml, PanEco, Russia) was performed for 12 h at 37 °C. The obtained suspension was centrifuged at 200*g* for 10 min at room temperature. The cell pellet was resuspended in DMEM/F12 supplemented with 2 mM l-glutamine (BioloT, Russia), 10% FBS (Thermo Fisher, USA), and penicillin/streptomycin (100 U/ml/100 µg/ml, PanEco, Russia) and seeded on Petri dishes.

#### UC-MSCs isolation

UC-MSCs were isolated as follows: umbilical cord samples were cut into pieces 2 cm long and washed from blood clots in phosphate buffer saline (PBS) supplemented with Diflucan (Pfizer, USA), gentamicin (50 µg/ml, PanEco, Russia), and penicillin/streptomycin (100 U/ml/100 µg/ml, PanEco, Russia). Then, longitudinal incisions were made so that the umbilical vein and umbilical arteries could be removed. Each sample was minced into small (~ 1 mm^3^) pieces with scissors and transferred into conical tubes. A fourfold volume of 0.2% collagenase NB4 (Serva, Germany), 0.005% hyaluronidase (Sigma, USA), and 10% Accutase (BD BioSciences, USA) in DMEM/F12 medium supplemented with 2 mM l-glutamine (BioloT, Russia) and 50 µg/ml gentamicin (PanEco, Russia) was added to each tube. Enzymatic dissociation was performed at 37 °C for 90 min with constant agitation on Mini Rocker-Shaker (Biosan, Latvia). The obtained suspension was passed through a 70-μm filter (Sigma, USA) and centrifuged at 100*g* for 7 min at room temperature. The cell pellet was resuspended in DMEM/F12 supplemented with 2 mM l-glutamine (BioloT, Russia), 10% FBS (Thermo Fisher, USA), and penicillin/streptomycin (100 U/ml/100 µg/ml, PanEco, Russia) and seeded on Petri dishes.

### Monolayers culturing

The cells were cultured in full growth medium consisting of DMEM/F12 supplemented with 2 mM l-glutamine (BioloT, Russia), 10% FBS (HyClone, USA), insulin–transferrin–sodium selenite (1:100, BioloT, Russia), bFGF (20 ng/ml, ProSpec, Israel), and gentamicin (50 μg/ml, PanEco, Russia) at 37 °C and 5% CO_2_. MSCs’ morphology and confluence were routinely checked with a phase-contrast microscope Primovert (Carl Zeiss, Germany). The media were replaced every other day, and the cells were passaged at 80% confluence. After 4th passage, the cells were used for spheroid formation and characterization (immunophenotyping and differentiation potential assessment). CM samples were collected on the 3rd day of culturing after 4th passage and stored at − 80 °C until further analysis.

### Spheroids formation

Cell spheroids (3000 cells per spheroid) were formed as previously described [25] and cultured likewise. Briefly, agarose non-adhesive microplates created with 3D Petri Dish molds (256 microwells per microplate) (Microtissues, USA) were transferred to the wells of 12-well culture plates, and cell suspension (5.1 × 10^6^ cells/ml) was placed into each microplate. The microplates were incubated for 1 h at 37 °C, 5% CO_2_ for cell setting, and then covered with full growth medium. CM samples were collected on the 3rd day of culturing and stored at − 80 °C until further analysis.

### Immunophenotyping

MSCs monolayers were treated with Versene solution (Invitrogen, USA) and 0.25% trypsin solution (Invitrogen, USA) to obtain single-cell suspensions. Cell spheroids were dissociated prior to the flow cytometry by gentle agitation on Mini Rocker-Shaker (Biosan, Latvia) in 1 ml TrypLE Express (Gibco, USA) per 250 spheroids for 15 min at 37 °C.

The obtained cell suspensions containing at least 1 million cells each were additionally washed in PBS to remove residual culture medium. The pellets were resuspended in PBS containing 1% FBS and aliquoted for subsequent staining with anti-human antibodies for CD105 (conjugated with PerCP-Cy™5.5), CD73 (conjugated with APC), CD90 (conjugated with FITC), CD44 (conjugated with PE), CD19, CD11beta, CD45, CD34, and HLA-DR (all negative markers conjugated with PE) (BD Stemflow™ hMSC Analysis Kit). The staining was performed according to manufacturer`s protocol. Cells stained with isotype control from kit (BD Stemflow™ hMSC Analysis Kit) and unstained cell suspension were used as controls. The antibodies were added in concentrations specified by the manufacturers, and the samples were incubated in the dark for 15 min at room temperature. The samples were then washed with PBS and analyzed on microfluidic cell sorter Sony SH800 (Sony Biotechnology, USA), recording at least 10,000 events per sample. The background level of fluorescence was determined using the unstained cell suspension, while antibody specificity was verified by comparing unstained suspensions with isotype controls. Each marker was subsequently compared to its respective isotype control.

### Differentiation potential

The isolated cells’ ability to maintain the multi-lineage differentiation was tested as follows: once 80% confluence was achieved, the medium was changed either to full growth medium (control) or to one of the differentiation media: osteogenic (StemPro™ Osteogenesis Differentiation Kit, ThermoFisher Scientific, USA), chondrogenic (StemPro™ Chondrogenesis Differentiation Kit, ThermoFisher Scientific, USA), or adipogenic (StemPro™ Adipogenesis Differentiation Kit, ThermoFisher Scientific, USA). The cells were cultured for 21 days, the media were changed every 3 days. Following 21 days of differentiation, the cells were washed in PBS and fixed in PFA (4%, pH 6.9, Sigma-Aldrich, Germany) for 20 min at 4 °C.

Osteogenic differentiation was evaluated using Alizarin red staining (Alizarin Red S, Sigma-Aldrich, Germany). The samples were washed in PBS, and a staining solution of 2% Alizarin Red S (pH 4.2) was added to the cells for 30 min.

Chondrogenic differentiation was evaluated using Alcian Blue staining (Sigma-Aldrich, Germany). The samples were washed with 1% acetic acid, then a 1% Alcian Blue staining solution in acetic acid (pH 2.5) was added, and cells were incubated overnight at 4 °C.

Adipogenic differentiation was evaluated using Oil Red O staining (Sigma-Aldrich, Germany). The samples were washed with 60% isopropanol and air-dried for 5 min; then, a 0.2% Oil Red O staining solution in 60% isopropanol was added to the cells for 30 min.

All samples were then thoroughly washed, and images were taken using the Primovert phase-contrast microscope (Carl Zeiss, Germany).

### Detection of cytokines and growth factors in MSCs-CM

Cytokines and growth factors in MSCs-CM samples were analyzed with the MILLIPLEX™ MAP Human Cytokine/Chemokine Magnetic Bead Panel (Merck Millipore, USA) utilizing xMAP technology (Luminex, USA). All CM samples contained cytokines and growth factors synthesized by 2.5 × 10^5^ cells per 1 ml.

Briefly, the 96-well plates were washed twice with the buffer provided in the kit. Then, the standards, controls, and samples were added to the wells according to the manufacturer's instructions. All samples were added in duplicate. Control (non-conditioned) culture medium was added as background to the standard and control wells, buffer was added to the sample wells, and ultrasound-pretreated magnetic beads were added to each well. While adding the beads, the tube containing them was vortexed every 30 s to avoid the particles settling and to ensure their even distribution into each well. The plates were then sealed and incubated overnight on Mini Rocker-Shaker (Biosan, Latvia) at 4 °C in the dark. The next day, the plates were washed twice with the buffer using a magnetic washer, so that the beads remained in the wells. The plates were then incubated sequentially with detection antibodies for 1 h and with streptavidin–phycoerythrin for 30 min at room temperature, with constant agitation on Mini Rocker-Shaker (Biosan, Latvia). Then, the plate was washed twice with the buffer on a magnetic washer. Drive fluid provided in the kit was added to each well, the plate was sealed and agitated on Mini Rocker-Shaker (Biosan, Latvia) for 5 min. Then, the plate was analyzed on a MagPix machine (Luminex, USA) with xPONENT (Luminex, USA) software.

### Monocyte isolation and differentiation into M0 macrophages

**Fig. 1:**
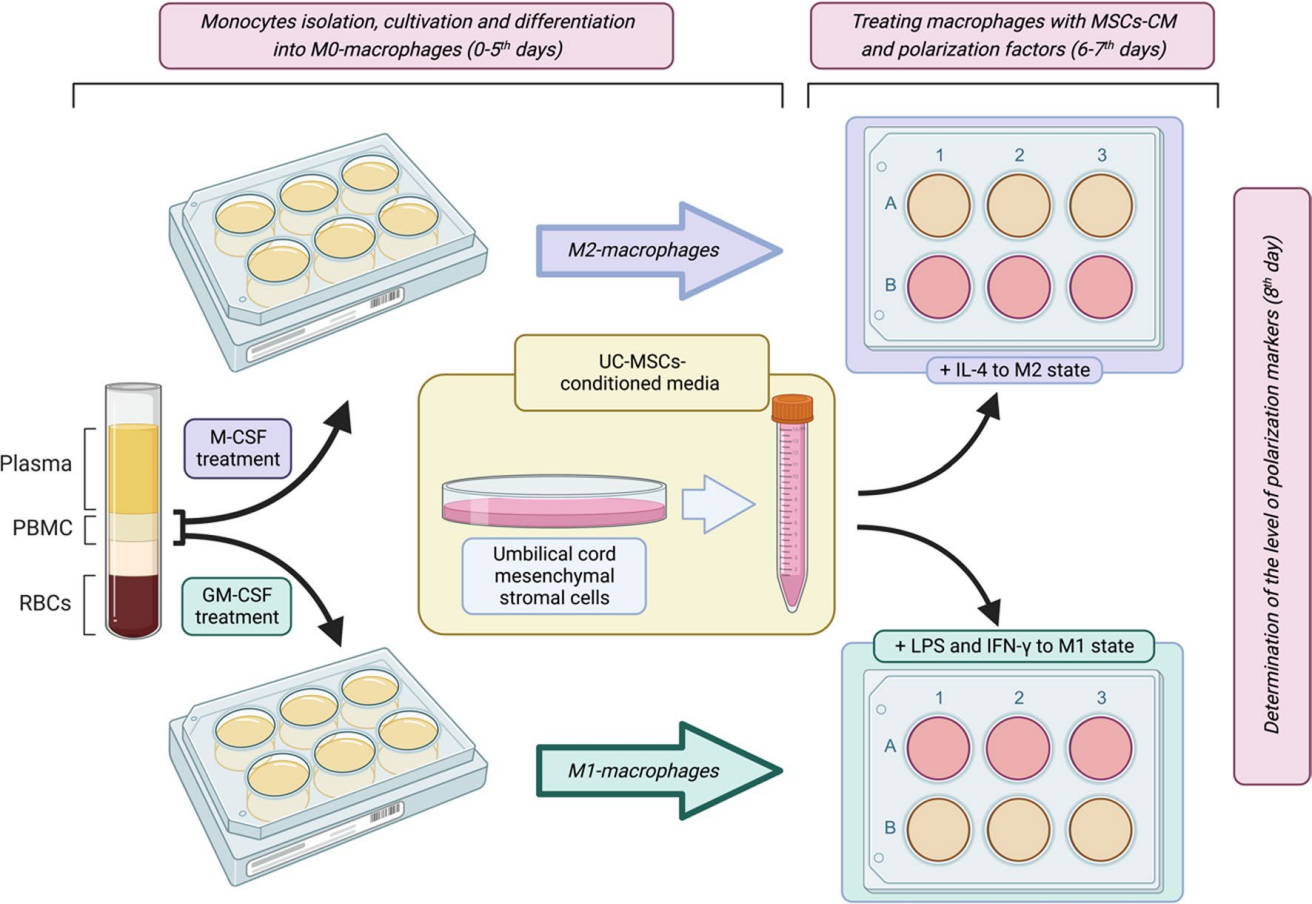
Scheme of the experiments with macrophages. PBMCs were seeded in culture plates and treated with relevant growth factors. On day 6, inducers of M1 or M2 macrophage polarization were added to GM-CSF- or M-CSF-treated macrophages, respectively. UM-MSCs-CM were added into half of the wells (colored pink). On day 8, the markers of M1 or M2 phenotypes were measured

Design of the following experiments including monocyte isolation, differentiation into M0 macrophages, and subsequent treatment with MSCs-CM is graphically presented in Fig. 1.

Monocytes were isolated from peripheral blood mononuclear cells (PBMCs). Blood was obtained from healthy volunteers. All donors have signed an informed consent form approved by the local ethics committee of Sechenov University.

Briefly, blood was mixed with PBS and layered onto Histopaque-1077 (Sigma-Aldrich, USA) for density gradient centrifugation at 400*g* for 40 min. After centrifugation, PBMCs layer was collected, cells were washed twice with PBS and plated at the density of 7.5–8.5 × 10^5^ PBMCs/cm^2^. Cells were cultured in RPMI complete medium consisting of RPMI-1640 (Corning, USA) supplemented with 10% autologous serum, penicillin (100 U/ml), and streptomycin (100 µg/ml) at 37 °C, 5% CO_2._ After 2 h of incubation, non-adherent cells were washed with PBS, and fresh RPMI complete medium containing 50 ng/ml GM-CSF or 50 ng/ml M-CSF (SCI-store, Russia) was added to adherent monocytes to differentiate them into M0 macrophages for the subsequent experiments with M1 or M2 cells, respectively. The medium was replaced with the fresh one on the 3rd day of culturing.

### Treating macrophages with MSCs-CM

On the 6th day of culturing, MSCs-CM were added to both GM-CSF- and M-CSF-treated macrophages in 1:1 ratio with RPMI complete medium (MSC-CM group). Fresh DMEM complete medium was used as a control (Control group). M-CSF-treated macrophages were additionally treated with 20 ng/ml IL-4 (Sigma-Aldrich, USA) to stimulate their M2 polarization (M2 group cultured with M-CSF) and GM-CSF-treated macrophages were additionally treated with 10 ng/ml LPS (Sigma-Aldrich, USA) and 50 ng/ml IFN-γ (Sigma-Aldrich, USA) to stimulate their M1 polarization (Fig. 1). Polarization markers were measured on the 8th day of culturing.

### Flow cytometry

On the 8th day of culturing, macrophages were incubated with accutase solution (Sigma-Aldrich, USA) for 5 min at 37 °C, 5% CO_2_ to obtain single-cell suspensions. The suspensions were added into RPMI complete medium (v/v = 1:8) and centrifuged at 300*g* for 10 min. After centrifugation, cell pellets were washed twice with PBS to remove residual accutase. Finally, cell pellets were resuspended in PBS containing 1% FBS and aliquoted for subsequent staining with anti-CD-206 antibodies conjugated with PE-Cy7 or with anti-CD86-FITC antibodies (Invitrogen, USA) according to the manufacturer’s protocol. After incubations with antibodies, the samples were washed with PBS and analyzed on microfluidic cell sorter Sony SH800 (Sony Biotechnology, USA) (Additional file 1: Figure S1).

### ELISA

On the 8th day of culturing, supernatants were collected from M1 macrophages and centrifuged at 300*g* for 10 min to exclude cell debris. The concentration of TNF-α, secreted by macrophages polarized toward the M1 state, was measured using an enzyme immunosorbent assay system according to the manufacturer's instructions (“Cytokine”, St. Petersburg, Russia). Optical density was measured using a Multiskan™ FC Microplate Photometer (ThermoFisher, USA).

### Statistical data analysis

Python (version 3.10) was used for data analysis. For multiplex analysis, deviations from the expected values of cytokine and growth factor concentrations were calculated as *z*-scores. Welch’s *t* test was used to compare differences between two samples. One-way analysis of variance (ANOVA) with Tukey’s post hoc test was applied for multiple comparison. The correlation analysis was conducted by Spearman’s rank test. *P* values less than 0.05 were considered statistically significant.

## Results

### The isolated cells correspond to the MSCs immunophenotype

To confirm that the isolated cells can be identified as MSCs, their immunophenotyping was performed In Methods, we determined the percentage of cells expressing the mesenchymal markers (CD105, CD73, CD90, and CD44) and the hematopoietic markers (CD19, CD11beta, CD45, CD34, and HLA-DR). It was demonstrated that the expression of negative markers was less than 1% in all cell populations. At the same time, positive markers were expressed in 97–99% of AT-MSCs, 95–99% of BM-MSCs, 91–98% of G-MSCs, 87–99% of PL-MSCs, and 98–100% of UC-MSCs (Additional file 1: Tables S1, S2; Additional file 1: Figures S2, S3).

### The isolated cells demonstrated trilineage differentiation capacities

The isolated cells demonstrated the ability of osteo-, chondro-, and adipogenic differentiation (Additional file 1: Figure S4). Alizarin Red staining determined the presence of calcium mineralization in the cell cultures, which corresponded to osteogenic differentiation; Alcian Blue staining determined the presence of mucopolysaccharides and glycosaminoglycans in the cell cultures, which corresponded to chondrogenic differentiation, and Oil Red O staining determined the presence of lipid droplets in the cell cultures, which corresponded to adipogenic differentiation.

The isolated cells were highly adhesive to the plastic and were actively proliferating, while maintaining spindle-shaped form. Together with their trilineage differentiation capacities and the immunophenotype corresponding to that of MSCs, the isolated cells can be considered MSCs.

### UC-MSCs-CM showed higher cytokine levels than MSCs-CM from other sources

**Fig. 2:**
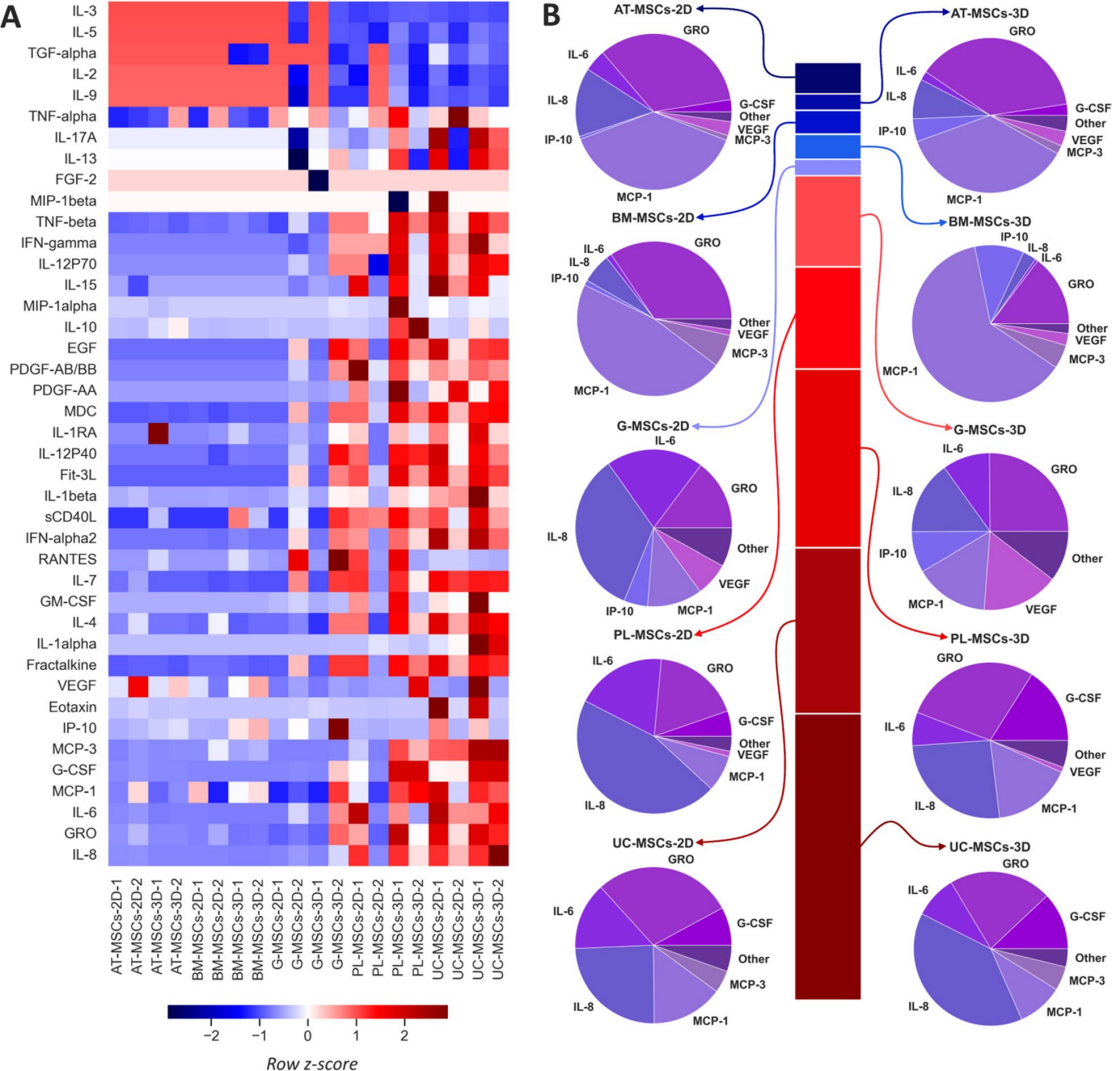
Cytokine profiles of AT-MSCs-CM, BM-MSCs-CM, G-MSCs-CM, PL-MSCs-CM, and UC-MSCs-CM from 2D and 3D cultures. **A** Heatmap of 41 cytokines in CM from five sources scaled to row z-score; **B** stacked bar chart showing the ratio between the volumes of secretomes of different CM and corresponding pie charts with cytokine profile structure

Expression of 41 cytokines (including chemokines) and growth factors in AT-MSCs-CM, BM-MSCs-CM, G-MSCs-CM, PL-MSCs-CM, and UC-MSCs-CM is presented as a heatmap reflecting their lower (blue shades) and higher (red shades) levels than the expected values for each sample (Fig. 2A). Since most of the substances listed on the left in the heatmap are cytokines (except for 8 growth factors), hereinafter we will refer these compounds as cytokines.

The rows were sorted in ascending order of total cytokine concentration in all samples in a row. The cytokines in the upper part of the heatmap were thus either detected in minor concentrations or not detected at all, which corresponds to the previous findings [26], and therefore are not of particular interest. At the same time, bottom cytokines demonstrated much higher values (Fig. 2A).

It is noteworthy that 2D and 3D cultures of MSCs from the same source exhibited identical prevailing cytokines with some difference in their ratio. At the same time, the prevailing cytokines were not identical for MSCs from different sources (Fig. 2B).

All CM except AT-MSCs-CM demonstrated higher cytokine levels in 3D cultures (Fig. 2B); however, due to the insufficient number of analyzed samples, this difference could not have statistical confirmation.

Out of five cell cultures, UC-MSCs demonstrated the highest cytokine levels in the conditioned media and were chosen for further analysis. Although two samples per group could not provide statistical significance for this choice either, we made our choice based on the stacked bar chart in Fig. 2B. Being most abundant in cytokines and growth factors both in 2D and 3D, UC-MSCs were of greatest research interest as CM source.

### UC-MSCs-CM demonstrated statistically significant difference in cytokine expression when cultured in 2D and 3D conditions

**Fig. 3:**
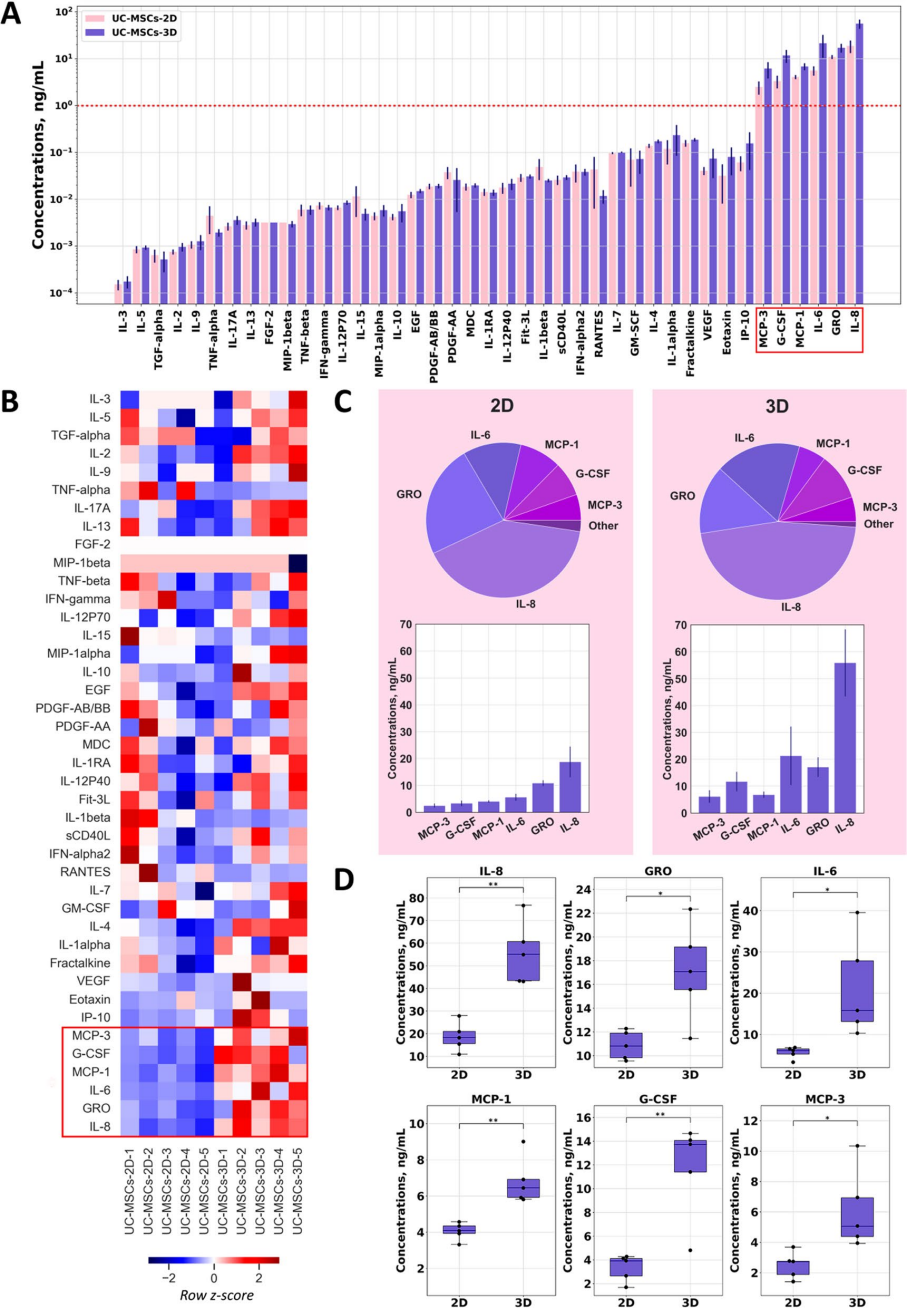
Cytokine concentrations in UC-MSCs-CM from 2D and 3D cultures. **A** Bar chart of cytokine absolute concentrations. The red horizontal dashed line separates cytokines with concentrations exceeding 1 ng/ml from other cytokines. Red rectangle borders six cytokines making major contribution to the UC-MSCs cytokine profile; **B** heatmap of 41 cytokines in UC-MSCs-CM scaled to row z-score. Red line borders six cytokines making major contribution to the UC-MSCs cytokine profile; **C** pie charts showing the percentage of six prevailing cytokines in UC-MSCs-CM from 2D and 3D cultures, and bar charts showing their absolute concentrations; **D** box plots showing differences in concentrations of the six prevailing cytokines in UC-MSCs-CM from 2D and 3D cultures. Statistical significance was determined using Welch’s *t* test; *p < 0.05; **p < 0.01

A series of independent experiments were performed to assess the expression of 41 cytokines in UC-MSCs-CM. The predominant number of cytokines was identified in small concentrations or not detected at all. However, the concentrations of IL-8, GRO, IL-6, MCP-1, G-CSF, and MCP-3 were higher than 1 ng/ml in all media (Fig. 3A).

The results are presented as a heatmap showing their lower (blue shades) and higher (red shades) levels than the expected values for each sample (Fig. 3B). *P* values for all cytokines, except FGF-2, in this study were less than 0.05 (Welch’s *t* test) and therefore were statistically significant.

The rows were equally sorted in ascending order of total cytokine concentration in all samples in a row, and the results were consistent with those presented in the previous section. Six bottom cytokines, namely the above-mentioned IL-8, GRO, IL-6, MCP-1, G-CSF, and MCP-3, were shown to make the major contribution to the cytokine profile of UC-MSCs (Fig. 3C).

Moreover, it was demonstrated that the cluster of the six prevailing cytokines is differentially expressed (*p* value is less than 0.05 according to the Welch’s *t* test) in UC-MSCs cultured in 2D and 3D conditions, showing significantly higher levels in 3D cultures (Fig. 3D).

### UC-MSCs-CM from 2D and 3D cultures promoted anti-inflammatory macrophages’ polarization despite their mostly pro-inflammatory cytokine profile

The ability of MSCs-CM to navigate polarization of macrophages was previously shown by several independent studies of mainly murine or THP-1 macrophages [4, 27], thus we focused on human monocyte-derived macrophages (MDM). UC-MSCs-CM were chosen among others as their cytokine profile contains the highest concentrations of cytokines (Figs. 2,3). Experiments were performed to study the effects of UC-MSCs-CM on the polarization of human macrophages toward pro-inflammatory M1 or anti-inflammatory M2 states.

**Fig. 4:**
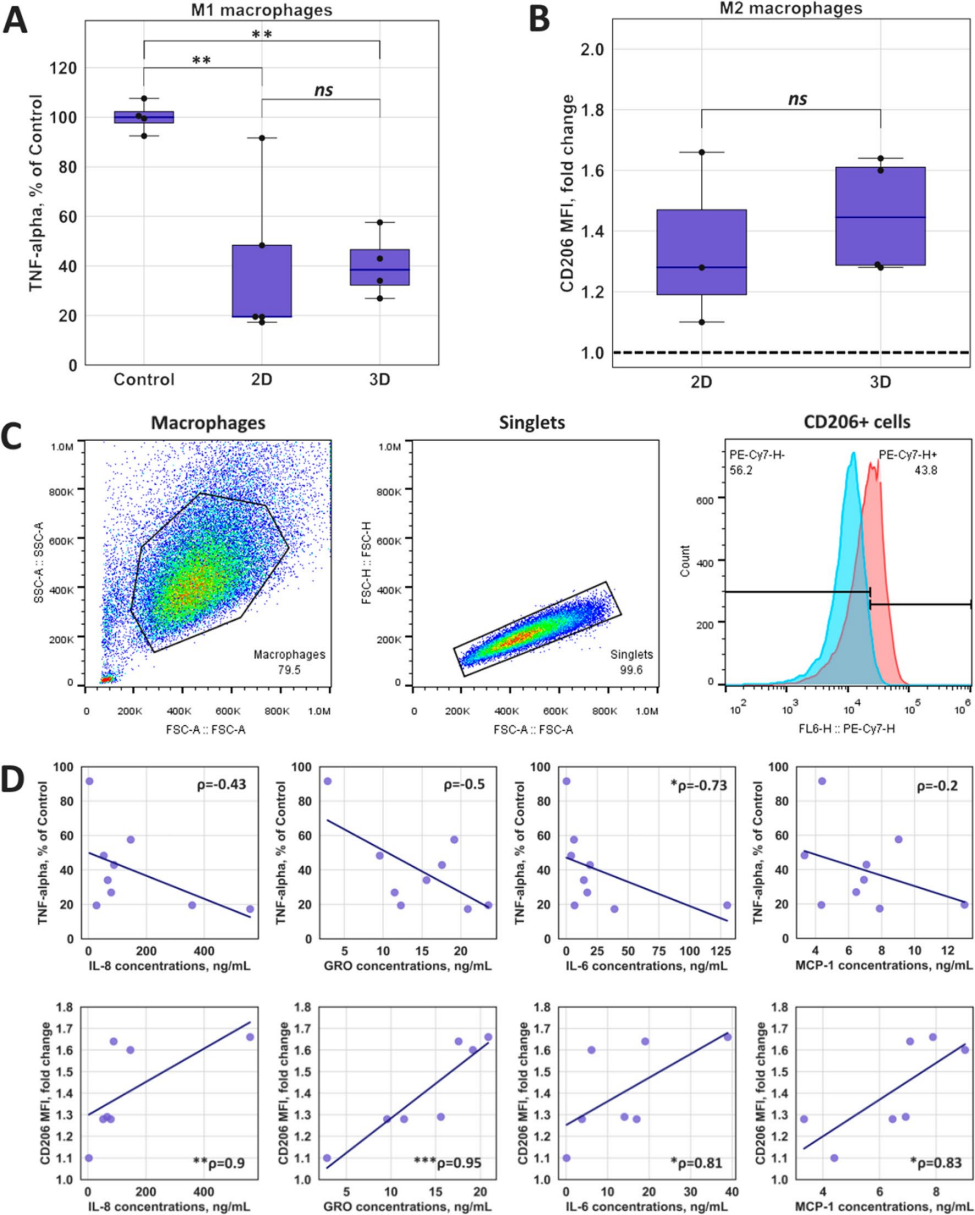
UC-MSCs-CM from 2D and 3D cultures navigated the polarization of human macrophages. **A** The concentration of TNF-α in culture media of MDM. MDM were treated with UC-MSCs-CM from 2D and 3D cultures, macrophages of control group were treated with DMEM complete media for 48 h. All cells were stimulated with LPS (10 ng/ml) and IFN-γ (50 ng/ml). The concentration of TNF-α in UC-MSCs-CM-treated cells is presented as percent of TNF-α concentration in control cells. Statistical significance was determined using a one-way ANOVA with Tukey’s post hoc test; **p < 0.01; ns = *p* > 0.05; **B** Surface expression of CD206 in untreated and UC-MSCs-CM-treated M2 macrophages evaluated by flow cytometry. Human macrophages of experimental group were treated with UC-MSCs-CM from 2D and 3D cultures, and macrophages of control group were treated with DMEM complete media for 48 h. All cells were additionally stimulated with IL-4 (20 ng/ml). Statistical significance was determined using Welch’s *t* test; ns = *p* > 0.05. Box plot demonstrates the fold change of median fluorescence intensity (MFI) of CD206 expression in UC-MSCs-CM-treated macrophages compared to untreated control which is represented by a dashed line; **C** Gating strategy used to identify CD206+ -positive macrophages. Forward scattering area (FSC-A) versus side scattering area (SSC-A) density plot was used to identify cells and exclude debris. FSC-Height versus FSC-A density plot was performed to analyze only single cells. Histograms represent fluorescence intensity of antiCD206-PE-Cy7 and allow to distinguish M0 and M2 macrophages; **D** Scatter plots showing correlation between macrophage markers and concentration of cytokines. Correlation coefficients (*ρ*) were calculated using Spearman’s rank test; *p < 0.05; **p < 0.01; ***p < 0.001

Despite the fact that cytokine profile of UC-MSCs-CM includes mainly pro-inflammatory molecules, incubation of M1 macrophages with UC-MSCs-CM from 2D or 3D cultures for 48 h led to a significant decrease in concentration of TNF-α which is the typical marker of M1 state comparing to control group (Fig. 4A). At the same time, we did not observe significant changes in CD86 expression after M1 polarization in the presence of three different UC-MSCs-CM (Additional file 1: Figure S1B). CD86 expression decreased in one case, while TNF-α secretion reduced significantly in media of all MDM incubated with UC-MSCs-CM. We did not observe the statistical difference between UC-MSCs-CM from 2D and 3D cultures either.

The ability of UC-MSCs-CM to polarize macrophages to anti-inflammatory M2 state was assessed via surface expression of macrophage mannose receptor (CD206), a canonical marker of M2 macrophages (Fig. 4B). Standard gating strategy was used to detect CD206-positive cells (Fig. 4C). M2 macrophages which were treated with IL-4 demonstrated higher CD206 expression after incubation with UC-MSCs-CM for 48 h compared to control. Similar to M1 phenotype, there was no statistical difference in marker expression between M2 macrophages treated with UC-MSCs-CM from 2D or 3D cultures (Fig. 4B). Taken together, these results suggest that despite their mostly pro-inflammatory cytokine profile UC-MSCs-CM are able to enhance expression of macrophages’ anti-inflammatory M2 marker CD206.

Spearman’s rank correlation test was performed to analyze the possible mechanism of UC-MSCs-CM effects on the macrophage polarization. Positive or negative correlations were observed between the concentrations of four cytokines (IL-6, IL-8, GRO, MCP-1) and M1 (TNF-α) or M2 (CD206) macrophage markers, respectively (Fig. 4D). Taking into consideration these estimations, the mechanisms and signaling pathways of UC-MSCs-mediated modulation of the anti-inflammatory activity of macrophages remain to be elucidated.

## Discussion

With the increasing interest toward cell-free therapies [28], secretome studies are acquiring great popularity. Several research groups reported secretion profiles of MSCs from different sources [26, 29, 30], and although obtained results are not identical, there are a few common patterns giving some general insights into MSCs’ secretome profiling.

Thus, Park and colleagues performed an analysis recognizing 120 cytokines and growth factors in BM-MSCs and reported 114 cytokines out of 120 to be detected in minor concentrations or not detected at all [26]. These findings are consistent with the results of the present study, since in several independent experiments with MSCs from different sources we observed that more than 80% of the media composition (ng/ml) accounted for 6–8 cytokines, while the contribution of the remaining cytokines did not exceed 10–20% (concentrations < 1 ng/ml) (Fig. 2).

Speaking of the cytokines and growth factors making the major contribution to the secretome of MSCs, Park and colleagues report six of them, namely IL-6, IL-8, MCP-1, VEGF, TIMP-2, and OPG [26]. In our study, we also found significant contribution of IL-6, IL-8, and MCP-1, while concentrations of VEGF were relatively low (< 1 ng/ml). Neither TIMP-2 nor OPG were evaluated in our study; however, we found significant contribution of MCP-3, G-CSF, and GRO.

3D culturing was repeatedly reported to provide a more physiological microenvironment for cell growth, leading to upregulated production of signaling molecules and enhancing their therapeutic potential [31–33]. Our research confirmed the cluster of the six prevailing cytokines to be differentially expressed in UC-MSCs cultured in 2D and 3D conditions, showing significantly higher levels in 3D cultures (Fig. 3). In the first series of experiments, all CM except AT-MSCs-CM also demonstrated higher cytokine levels in 3D cultures (Fig. 2B); however, due to the lack of analyzed samples, this difference could not be considered statistically significant.

The immunomodulatory effect of MSCs on macrophage activity is well documented: MSCs induce the shift of macrophages polarization from the classically activated pro-inflammatory M1 to alternatively activated anti-inflammatory M2 state, which promotes regeneration. However, the causes of MSC-mediated macrophage phenotype modulation are still under investigation. Using a human monocytic cell line THP-1, Vasandan and colleagues have demonstrated MSCs’ ability to attenuate inflammatory M1 state with concomitant shift toward alternative M2 activation and further enhancement of M2 macrophages secretory activity. The authors showed that the immunomodulatory effect of MSCs on macrophages was mediated by prostaglandin E2 (PGE2) secretion [34]. Recent studies with different types of MSCs and macrophages have shown that extracellular vesicles (EVs) secreted by MSCs reduce M1 polarization and promote macrophage activation into a reparative M2 state in a variety of settings [27, 35]. Chemokines CCL2 (MCP-1) and CXCL12 secreted by MSCs were shown to cooperatively activate immunosuppressive IL-10 + tissue macrophages to mitigate the negative consequences of intestinal pathologies [36].

As a result of all studies, the general consensus is that MSCs-dependent macrophage control is mainly due to the secreted liquid-phase factors such as EVs, prostaglandins, cytokines, and growth factors rather than MSC-to-macrophage contacts [1]. Therefore, the number of studies using MSCs’ conditioned media instead of MSCs’ cultures has recently increased. Jin and colleagues showed the anti-inflammatory effect of MSCs-CM in vivo and found the ability of these media to inhibit the activation of murine RAW264.7 macrophages by lipopolysaccharide into M1 state in vitro [37]. Murine bone marrow MSCs-CM were shown to promote the polarization of murine bone marrow-derived macrophages (BMDM) into M2 state [38] reducing the negative consequences of a number of pathologies, for example, the development of aortic aneurysm [39].

In the present study, we demonstrated that the UC-MSCs-CM promoted human MDM polarization into M2 state, increasing the expression of CD206 receptor, and inhibited the anti-inflammatory activity of M1 phenotype, reducing secretion of pro-inflammatory cytokine TNF-α (Fig. 4). At the same time, we did not observe correlation between expression of CD86 and TNF-α secretion in M1 cells incubated with UC-MSCs-CM (Additional file 1: Figure S1B). Smith and colleagues demonstrated earlier that within 48 h of observation, small quantities of IL-4/IL-13 stimuli could enhance CD86 expression in LPS/IFN-ꝩ-stimulated mouse BMDM [40]. Moreover, there is evidence that surface marker expression does not always directly correlate with cytokine production by macrophages [41].

The simplified division of macrophages into M1 and M2 phenotypes does not take into account the diversity of polarization subtypes of these cells. M2 macrophages can be divided into M2a, M2b, M2c, and M2d subtypes based on transcriptional changes induced by different stimuli [42]. For example, CD86 is expressed in M2b macrophages and is considered as a marker for this subtype. At the same time, M2b cells do not express CD206 or CD163, which are typical markers of M2 phenotype, but secrete IL-10 like other M2 cells. Depending on the composition of the conditioned media, one can expect a wide variety of macrophage phenotypes according to the polarization scale.

No statistical difference between UC-MSCs-CM from 2D and 3D cultures was observed when assessing their anti-inflammatory effect on M2 and M1 macrophages. This outcome may be explained by the fact that although 3D UC-MSCs cultures have shown higher levels of cytokines, this difference could not provide a significant shift in macrophages polarization. On the other hand, the contribution of other components of MSCs secretome (EVs or cytokines and growth factors beyond those analyzed in this study) might be significant enough to mitigate the difference between CM from 2D and 3D cultures when assessing their effect on macrophage polarization. For example, the panel used by Miranda and colleagues included LIF, I-309, 6CKine, and SCF, which were reported to be elevated in 3D UC-MSCs cultures, while IL-21 was reported to be elevated in 2D cultures [43]. PGE2, an important player in MSC-mediated macrophages polarization toward M2 phenotype, was reported to be elevated in BM-MSCs 3D cultures by Ylöstalo and colleagues [44]. However, cell spheroids studied in this paper were much bigger than in our study (25 000 cells per spheroid) and therefore may represent a different secretome pattern. Speaking about EVs derived from 2D and 3D MSCs cultures and their effect on macrophages polarization, there is little research on this topic and data are rather controversial. For example, no difference in macrophage polarization was reported for BM-MSCs-derived EVs from 2D and 3D cultures [45]. At the same time, EVs from human umbilical cord blood MSCs cultured in 3D conditions were reported to have an enhanced M2 polarization ability on macrophages [46].

The anti-inflammatory cytokines like IL-1RA, IL-10, and IL-13 were detected in UC-MSCs-CM at extremely low concentrations (1–20 pg/ml) and therefore could not affect MDM polarization. At the same time, the prevailing components found in UC-MSCs-CM (Fig. 3) are considered mostly pro-inflammatory, which contradicts MSCs-CM anti-inflammatory properties. Indeed, MCP-3, MCP-1, GRO, and IL-8 are chemokines attracting immune cells, G-CSF is a factor promoting their growth, and IL-6 is a pleiotropic cytokine exhibiting both pro-inflammatory and anti-inflammatory functions.

While there is lacking evidence on the effect of MCP-3 on macrophage polarization, reports on the effect of MCP-1 (CCL2) are rather controversial. There is evidence of MCP-1 being an inducer of M2 macrophages [47]. However, the impact of MCP-1 on macrophage polarization seems to be context-dependent [48]. Speaking of G-CSF, it was reported to decrease M1/M2 ratio in bone marrow and peripheral blood from healthy donors [49].

IL-8, despite being considered a pro-inflammatory chemokine, was surprisingly reported to exhibit properties beneficial for M2 macrophages polarization [50]. IL-8 is highly secreted by tumor-associated macrophages and through a complex cytokine network can differentiate macrophages into immune-suppressing M2-like phenotype, which aggravate inflammatory breast cancer (IBC) progression. IBC cells in turn were reported to secrete factors that recruit monocytes and induce THP-1 cells and primary monocytes differentiation to M2-like polarized macrophages [51]. The authors supposed that IL-8 and GRO presented in CM of IBC cells at concentrations of about 20 pg/ml were responsible for monocyte activation. It is noteworthy that these concentrations are much lower than those found in UC-MSCs-CM in our study (about 5–20 ng/ml). Neurotensin/IL-8 pathway was also demonstrated to orchestrate local inflammatory response and tumor invasion by inducing M2 polarization of tumor-associated macrophages in mouse hepatocellular carcinoma [52]. The authors revealed that THP-1 cells culturing in IL-8-supplemented medium (5 ng/ml) demonstrated a significant increase in M2 markers, while the percentage of M1 phenotype decreased.

IL-6 is pleiotropic cytokine that has both pro- and anti-inflammatory functions and participates in immune regulation and tissue regeneration. Its role in stimulation of macrophages polarization into M2 state is well documented in many studies with different types of macrophages [53, 54]. The mechanism of this process is also proposed. IL-6 classical signaling via the membrane-bound receptor IL-6R controls IL-4R expression, while IL-4R is a part of both receptor complex for cytokines IL-4 and IL-13 inducing M2 differentiation [55]. In 2014, two studies independently revealed this new and surprising anti-inflammatory function of IL-6. Mauer and colleagues demonstrated that inactivation of IL-6R/IL-6 signaling in myeloid cells of experimental animals attenuates obesity-induced inflammation and insulin resistance by promoting M2 macrophage alternative activation. As compared to IL-4 treatment, treatment with IL-4 (1 or 10 ng/ml) and IL-6 (50 ng/ml) synergistically enhanced the expression of M2 macrophage-associated markers in BMDM from wild-type but not from IL-6R-deficient mice [56]. At the same time Fernando and colleagues revealed that co-treatment of BMDM or MDM with IL-6 (10 ng/ml, 48 h) and IL-4 + IL-13 (20 ng/ml) resulted in spontaneous release of anti-inflammatory IL-10 cytokine and enhanced expression of markers typical of mice or human M2 macrophages, while in the presence of IFNꝩ, IL-6 promoted the production of pro-inflammatory IL-1β and TNF-α by macrophages. The authors speculated that IL-6 could enhance the phenotype to which a macrophage has committed [57].

A large-scale screening enrolling more donors could have given a better insight into the heterogeneity of MSCs-CM samples and promote better understanding of the correlations between the effect of MSCs-CM on macrophages and the concentrations of cytokines/growth factors. Moreover, it is noteworthy that the interplay between MSCs and macrophages is not limited to the 41 cytokines and growth factors analyzed in our study. Contribution of other factors (for example, PGE2) and EVs cannot be ruled out, giving scope for further comprehensive research. In our future studies, we plan to further elucidate the impact of the MSCs-CM cytokines on M2 macrophage polarization as well as to study the contribution of EVs to this finely tuned process.

## Conclusions

In our study, we have provided an overview of cytokines and growth factors in conditioned media from adipose tissue-, bone marrow-, gingiva-, placenta-, and umbilical cord-derived MSCs cultured in 2D and 3D conditions. We have found that umbilical cord-derived MSCs conditioned media demonstrated the highest cytokine and growth factor levels and despite mostly pro-inflammatory cytokine profile were able to increase the expression of human macrophages’ polarization marker CD206 in the M2 anti-inflammatory phenotype and reduce the secretion of the pro-inflammatory cytokine TNF-α by macrophages in M1 state.

## Supplementary Information

**Additional file 1: Figure S1.** Flow cytometry detection of MDM polarization markers.** Table S1.** Immunophenotype of the MSCs isolated from human adipose tissue, bone marrow, gingiva, placenta, and umbilical cord (2D cultures).** Table S2.** Immunophenotype of the MSCs isolated from human adipose tissue, bone marrow, gingiva, placenta, and umbilical cord (3D cultures).** Figure S2.** Characterization of AT-MSCs, BM-MSCs, G-MSCs, PL-MSCs, and UC-MSCs by flow cytometry (2D cultures).** Figure S3.** Characterization of AT-MSCs, BM-MSCs, G-MSCs, PL-MSCs, and UC-MSCs by flow cytometry (3D cultures).** Figure S4.** The differentiation potential of the isolated cells.

## Data Availability

The datasets used and/or analyzed during the current study are available from the corresponding author on reasonable request.

## Abbreviations

ANOVA: Analysis of variance
AT-MSCs: Adipose tissue-derived multipotent mesenchymal stromal cells
bFGF: Basic fibroblast growth factor
BMDM: Bone marrow-derived macrophages
BM-MSCs: Bone marrow-derived multipotent mesenchymal stromal cells
CM: Conditioned media
DMEM: Dulbecco’s Modified Eagle’s Medium
EVs: Extracellular vesicles
FBS: Fetal bovine serum
G-CSF: Granulocyte colony-stimulating factor
G-MSCs: Gingiva-derived multipotent mesenchymal stromal cells
IL: Interleukin
IP-10: Interferon-γ-induced protein 10
MCP: Monocyte chemotactic protein
MDM: Monocyte-derived macrophages
MFI: Mean fluorescence intensity
MSCs: Multipotent mesenchymal stromal cells
PBMCs: Peripheral blood mononuclear cells
PBS: Phosphate buffer saline
PGE2: Prostaglandin E2
PL-MSCs: Placenta-derived multipotent mesenchymal stromal cells
SDF-1: Stromal cell-derived factor-1
TIMP-2: Tissue inhibitor of metalloproteinases 2
TNF-α: Tumor necrosis factor alpha
OPG: Osteoprotegerin
UC-MSCs: Umbilical cord-derived multipotent mesenchymal stromal cells
VEGF: Vascular endothelial growth factor
