# Supplementary material for: MSCs’ conditioned media cytokine and growth factor profiles and their impact on macrophage polarization
Source: Stem Cell Res Ther. 2023 May 25;14:142. doi: 10.1186/s13287-023-03381-w (PMC10214600; doi:10.1186/s13287-023-03381-w)
Supplement: Supplementary file 1 — Additional file 1: Figure S1. Flow cytometry detection of MDM polarization markers. Table S1. Immunophenotype of the MSCs isolated from human adipose tissue, bone marrow, gingiva, placenta, and umbilical cord (2D cultures). Table S2. Immunophenotype of the MSCs isolated from human adipose tissue, bone marrow, gingiva, placenta, and umbilical cord (3D cultures). Figure S2. Characterization of AT-MSCs, BM-MSCs, G-MSCs, PL-MSCs, and UC-MSCs by flow cytometry (2D cultures). Figure S3. Characterization of AT-MSCs, BM-MSCs, G-MSCs, PL-MSCs, and UC-MSCs by flow cytometry (3D cultures). Figure S4. The differentiation potential of the isolated cells. [file 13287_2023_3381_MOESM1_ESM.docx]

**Supplementary information**

**MSCs’ conditioned media cytokine and growth factor profiles and their impact on macrophage polarization**

Maria Peshkova ^1,2,3^, Alexander Korneev* ^2,3,4^, Shakir Suleimanov* ^2,3^, Irina I. Vlasova ^2^,

Andrey Svistunov ^5^, Nastasia Kosheleva ^2,3,6^, and Peter Timashev ^1,2,3^

^1^ World-Class Research Center “Digital Biodesign and Personalized Healthcare”, Sechenov University, 119991 Moscow, Russia

^2^ Institute for Regenerative Medicine, Sechenov University, 119991 Moscow, Russia

^3^ Laboratory of Clinical Smart Nanotechnologies, Sechenov University, 119991 Moscow, Russia

^4^ Laboratory of the Polymers Synthesis for Medical Applications, Sechenov University, 119991 Moscow, Russia

^5^ Sechenov First Moscow State Medical University, 119991 Moscow, Russia

^6^ FSBSI Institute of General Pathology and Pathophysiology, 125315 Moscow, Russia

* these authors contributed equally

** corresponding author: Peter Timashev, PhD., World-Class Research Center "Digital biodesign and personalized healthcare", Sechenov University, Moscow, Russia. Email: [timashev_p_s@staff.sechenov.ru](mailto:timashev_p_s@staff.sechenov.ru)

**Figure S1.** Flow cytometry detection of MDM polarization markers.


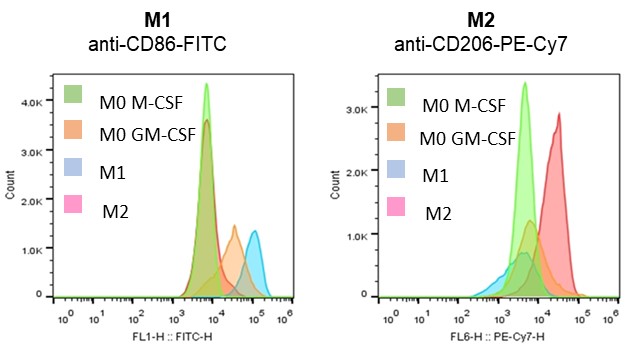


A. Histograms represent fluorescence intensity of M0, M1 and M2 macrophages. Cells cultured with GM-SCF (50 ng/ml) were treated by LPS (10 ng/ml) and IFN-ꝩ (50 ng/ml), cells cultured with M-CSF (50 ng/ml) were treated by IL-4 (10 ng/ml). In two days of incubation with inducers, macrophages were detached by accutase and stained with anti-CD86-FITC or anti-CD206-PE-Cy7 antibodies.

B. MSCs’ conditioning media (CM) were added to the GM-CSF-macrophages simultaneously with LPS and IFN-ꝩ (n=3). Histogramms show fluorescence intensity of anti-CD86-FITC antibodies in control M1 cell suspension (blue) and in M1 cells cultured with three different MSC’s CM (violet) after 2 days of incubation.

TNF- α: Concentration of pro-inflammatory cytokine TNF-α in culture media of M1 Control and M1 cells incubated in the presence of MSC’s-CM.

**Table S1.** Immunophenotype of the MSCs isolated from human adipose tissue, bone marrow, gingiva, placenta, and umbilical cord (2D cultures).

|  |  | **AT-MSCs** | **BM-MSCs** | **G-MSCs** | **PL-MSCs** | **UC-MSCs** |
| --- | --- | --- | --- | --- | --- | --- |
|  | **Markers** | ***Expression, %*** | | | | |
| ***Positive*** | CD73 | 97.97 | 99.99 | 97.81 | 87.86 | 98.35 |
|  | CD44 | 99.95 | 99.98 | 98.84 | 99.99 | 100.00 |
|  | CD90 | 99.41 | 95.30 | 96.83 | 98.37 | 99.95 |
|  | CD105 | 99.21 | 99.53 | 91.27 | 91.61 | 99.57 |
| ***Negative*** | CD45 | 0.06 | 0.23 | 0.21 | 0.02 | 0.00 |
|  | CD34 | 0.06 | 0.23 | 0.21 | 0.02 | 0.00 |
|  | CD19 | 0.06 | 0.23 | 0.21 | 0.02 | 0.00 |
|  | CD11β | 0.06 | 0.23 | 0.21 | 0.02 | 0.00 |
|  | HLA-DR | 0.06 | 0.23 | 0.21 | 0.02 | 0.00 |

**Table S2.** Immunophenotype of the MSCs isolated from human adipose tissue, bone marrow, gingiva, placenta, and umbilical cord (3D cultures).

|  |  | **AT-MSCs** | **BM-MSCs** | **G-MSCs** | **PL-MSCs** | **UC-MSCs** |
| --- | --- | --- | --- | --- | --- | --- |
|  | **Markers** | ***Expression, %*** | | | | |
| ***Positive*** | CD73 | 91.45 | 96.42 | 95.20 | 92.60 | 94.46 |
|  | CD44 | 99.99 | 100.00 | 96.10 | 99.97 | 100.00 |
|  | CD90 | 99.41 | 93.06 | 96.91 | 99.56 | 97.13 |
|  | CD105 | 95.12 | 98.07 | 96.35 | 96.13 | 94.75 |
| ***Negative*** | CD45 | 0.44 | 0.85 | 0.22 | 0.25 | 0.75 |
|  | CD34 | 0.44 | 0.85 | 0.22 | 0.25 | 0.75 |
|  | CD19 | 0.44 | 0.85 | 0.22 | 0.25 | 0.75 |
|  | CD11β | 0.44 | 0.85 | 0.22 | 0.25 | 0.75 |
|  | HLA-DR | 0.44 | 0.85 | 0.22 | 0.25 | 0.75 |

**Figure S2.** Characterization of AT-MSCs, BM-MSCs, G-MSCs, PL-MSCs, and UC-MSCs by flow cytometry (2D cultures).

The cells (passage 4) were stained for the specific surface markers as described in Materials and Methods section. Expanded cells demonstrated expression of CD73, CD44, CD90, CD105 and were negative for CD45, CD34, CD19, CD11beta, and HLA-DR. Isotype controls (green) were run for comparison.

**Figure S3.** Characterization of AT-MSCs, BM-MSCs, G-MSCs, PL-MSCs, and UC-MSCs by flow cytometry (3D cultures).

Cell spheroids were dissociated, and obtained cell suspensions were stained for the specific surface markers as described in Materials and Methods section. Сells demonstrated expression of CD73, CD44, CD90, CD105 and were negative for CD45, CD34, CD19, CD11beta, and HLA-DR. Isotype controls (green) were run for comparison.

**Figure S4.** The differentiation potential of the isolated cells


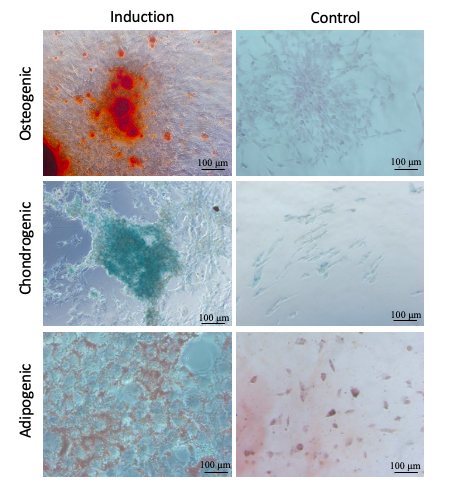


Induction: 21st day of culturing in differentiation media; control: 21st day of culturing in full growth medium; osteogenic differentiation: calcium deposits are stained red with Alizarin Red; osteogenic differentiation: mucopolysaccharides and glycosaminoglycans are stained blue with Alcian Blue; adipogenic differentiation: lipid droplets are stained red with Oil Red O. Phase-contrast microscopy; scale bar = 100 µm.
